# Analysis of genetic diversity in Brown Swiss, Jersey and Holstein populations using genome-wide single nucleotide polymorphism markers

**DOI:** 10.1186/1756-0500-5-161

**Published:** 2012-03-22

**Authors:** Melkaye G Melka, Flavio S Schenkel

**Affiliations:** 1 Centre for Genetic Improvement of Livestock, Department of Animal and Poultry Science, University of Guelph, 50 Stone Road East, Ontario, N1G 2W1, Canada

**Keywords:** Genetic diversity, SNPs, Genome-wide

## Abstract

**Background:**

Studies of genetic diversity are essential in understanding the extent of differentiation between breeds, and in designing successful diversity conservation strategies. The objective of this study was to evaluate the level of genetic diversity within and between North American Brown Swiss (BS, n = 900), Jersey (JE, n = 2,922) and Holstein (HO, n = 3,535) cattle, using genotyped bulls. GENEPOP and FSTAT software were used to evaluate the level of genetic diversity within each breed and between each pair of the three breeds based on genome-wide SNP markers (n = 50,972).

**Results:**

Hardy-Weinberg equilibrium (HWE) exact test within breeds showed a significant deviation from equilibrium within each population (*P* < 0.01), which could be a result of selection, genetic drift and inbreeding within each breed. Hardy-Weinberg test also confirmed significant heterozygote deficit in each breed over several loci. Moreover, results from population differentiation tests showed that the majority of loci have alleles or genotypes drawn from different distributions in each breed. Average gene diversity, expressed in terms of observed heterozygosity, over all loci in BS, JE and HO was 0.27, 0.26 and 0.31, respectively. The proportion of genetic diversity due to allele frequency differences among breeds (F_st_) indicated that the combination of BS and HO in an ideally amalgamated population had higher genetic diversity than the other pairs of breeds.

**Conclusion:**

Results suggest that the three bull populations have substantially different gene pools. BS and HO show the largest gene differentiation and jointly the highest total expected gene diversity compared to when JE is considered. If the loss of genetic diversity within breeds worsens in the future, the use of crossbreeding might be an option to recover genetic diversity, especially for the breeds with small population size.

## Background

The importance of genetic diversity in livestock is directly related to the need for genetic improvement of economically important traits as well as to facilitate rapid adaptation to potential changes in breeding goals [1]. Estimates of effective population size in commercial dairy populations, including Brown Swiss, Holstein and Jersey are decreasing at alarming rates to be of serious concern to the livestock industry [2]. Recently pedigree-based studies revealed increasing rates of inbreeding and coancestry in Canadian Jersey and Holstein populations [3]. Studies of genetic diversity are useful to the understanding of evolution of breeds, gene pool development and the level of differentiation among breeds [1,4,5]. Such studies are quite important for prioritizing conservation of breeds with critically low levels of diversity.

Hardy-Weinberg Equilibrium (HWE) states that in a large random mating population with no selection, mutation, or migration, the allele frequencies and the genotype frequencies are constant from generation to generation, and, hence, a simple relationship between the allele frequencies and the genotype frequencies exists [6]. The theory of HWE has played an important role in the development of population genetics, and has frequently been used as a basis for genetic inferences [7].

Tests for departures from Hardy-Weinberg proportions are often used to check on random mating in populations, and the deviations from the expected frequency of homozygotes are used to estimate inbreeding coefficients [8]. The same approach was used for estimating the inbreeding coefficient of a population by calculating the excess of homozygotes with respect to Hardy-Weinberg equilibrium expectations [9]. The role that the variance due to differences in gene frequencies among subpopulations play in the total genotypic frequencies from amalgamating subpopulations has been demonstrated by several studies [10,11]. Fixation indices (F_is_, F_it_ and F_st_) are the most widely used parameters for studying the genetic differentiation of populations. These indices have been originally defined in terms of the correlations of two uniting gametes [12-14]. Accordingly, F_it_ is the correlation between uniting gametes that generate an individual relative to the gametes of the total population. F_is_ is the average over all subpopulations of the correlation between uniting gametes that generate an individual relative to the gametes of their own subpopulation. F_st_ is the correlation between random gametes within subpopulations, relative to gametes of the total population. For example, in this study, an ideally amalgamated population of Brown Swiss and Jersey bulls would have each breed as a subpopulation. Furthermore, the relationship between fixation indices and measures of identity by decent have been illustrated in previous studies [15,16].

Fixation indices can also be formulated entirely in terms of the allelic and genotypic frequencies in the population [11,17,18]. In this case the fixation indices can be expressed in terms of ratios of heterozygosities. The F_st_ is equal to 0 when the same allele is fixed in all populations [11]. Allelic and genotypic frequencies may fluctuate because of finite subpopulation sizes or random variation in evolutionary forces [6]. In view of different factors affecting probabilities of gene identity in subdivided populations, the fixation indices were redefined in terms of the observed and expected heterozygosity based on allelic and genotypic frequencies in a population [17]. In addition, measures of inter-population gene differences and coefficients of gene differentiation (D_st_ and G_st_, respectively) have been extensively used to describe the level of genetic diversity [11,17].

The objective of this study was to assess the status of genetic diversity within and between BS, JE and HO breeds, using bulls genotyped with a dense SNP marker map through detailed analyses carried out via GENEPOP and FSTAT software.

## Methods

Genome-wide SNP data for the three breeds were received from the Animal Improvement Programs Laboratory, USDA (Beltsville, MD, USA) in November 2009. The data consisted of 900, 2,922 and 3,535 Brown Swiss (BS), Jersey (JE) and Holstein (HO) bulls, respectively, all genotyped with the Illumina BovineSNP50K BeadChip (Illumina Inc., San Diego, CA) as part of the North American collaboration in genomic prediction in dairy cattle [19]. Genotypes for a total of 50,972 SNPs were available for the analyses, which included all the SNPs with useable calls, without any exclusion due to minor allele frequency or correlation between SNPs. The bulls included in the analyses represented a sample of most BS and JE proven/sampled bulls in North America and a large sample of proven HO bulls in North America.

### Genetic diversity analysis

Estimates of genetic diversity and statistical analyses were performed using the software GENEPOP, version 4.0 [20]. The exact tests for deviations from HWE [9] were also performed using the GENEPOP package. GENEPOP uses a Markov Chain (MC) algorithm (dememorization = 10,000, batches = 100, and iterations per batch = 5,000) to estimate the P-value of the exact HWE tests [20]. Significance levels were calculated per locus, per breed, and over all loci and pairs of breeds combined. Genetic diversity within breeds was also measured as the frequency of private alleles (PA, breed-specific alleles), the observed heterozygosity (H_o_), and the expected heterozygosity (H_e_) under HWE. The significance of breed differences was tested using the exact test of population differentiation in GENEPOP software based on allele frequencies.

Genetic differentiation between breeds was also estimated using the F_st_ coefficient proposed by Wright [18] and computed by GENEPOP.

The software FSTAT [21] was used to compute F-statistic [12], and to test them using randomisation methods. The F_st_ was estimated by a “weighted” analysis of variance [21]. The most common computational formula for F_st_ is:

(1) $$F_{\mathrm{st}}=\frac{\delta_{p}{}^{2}}{p(1-p)}$$

Where: δ_p_^2^ the sample variance of allele frequencies over populations [11]. F_st_ can therefore be described as the amount of allele frequency variance in a sample relative to the maximum possible variance. F_st_ can also be defined as follows [14]:

(2) $$F_{\mathrm{st}}=\frac{F_{\mathrm{it}}-F_{\mathrm{is}}}{1-F_{\mathrm{is}}}$$

Where: F_it_ is the correlation between uniting gametes that generate an individual, relative to the gametes of the total population; F_is_ is the average, over all subpopulations, of the correlation between uniting gametes that generate an individual relative to those of their own subpopulation.

The amount of heterozygosis (Y_t_) in the total population was also defined regardless of structure of the population, in terms of total population gene frequency (q_t_) [14]:

(3) $$Y_{t}=2q_{t}(1-q)(1-F_{\mathrm{it}})$$

Indirect estimates of gene flow were implemented in FSTAT [21] according to the method demonstrated by [22]. The effective number of migrants (N_m_) was estimated, assuming the n-island model of population structure, on the basis of the relationship:

(4) $$F_{\mathrm{st}}=\frac{1}{1+4Nma}\;\text{,}\;where\;\alpha ={\left(\frac{n}{n-1}\right)}^{2}$$

Furthermore, FSTAT was used to calculate inter-population gene differences and coefficients of gene differentiation that are either dependent (D_st_ and G_st_) or independent (D_st_’ and G_st_’) of the number of subpopulations [11]. D_st_ is the average gene diversity between subpopulations. The gene diversity in the total population is equivalent to the sum of gene diversities within each subpopulation. Coefficient of gene differentiation (G_st_) was computed as the ratio of D_st_ to the total population diversity.

## Results

**Figure 1:**
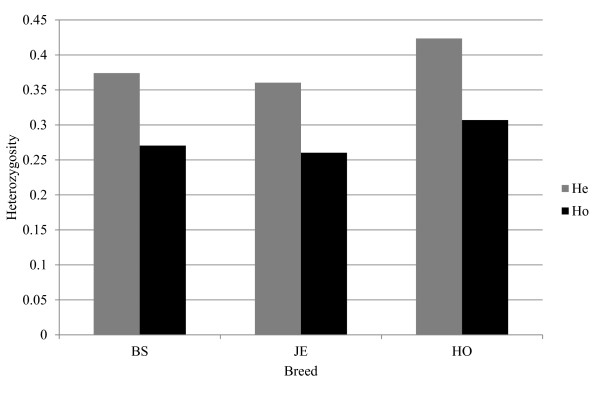
**Average gene diversity expressed in terms of heterozygosity expected under random mating (H**_**e**_**) and observed heterozygosity (H**_**o**_**).**

The exact test for Hardy-Weinberg Equilibrium (HWE) within breeds showed a significant deviation in each breed (*P* < 0.01). Moreover, results of the exact test for HWE showed lower observed heterozygosity (H_o_) than expected heterozygosity (H_e_) in each breed (Figure 1). The Holstein bull population showed the highest average marker diversity between individuals within breeds in terms of H_e_ compared to BS and JE breeds (0.31, 0.27, and 0.26, respectively). Jersey showed higher percentage of loci with fixed alleles followed by BS and HO (Table 1). The HWE test has also confirmed significant heterozygote deficit (≥90%) in each population over several loci.

**Table 1:** Average genotype and allele frequencies and percentage of loci with fixed alleles in Brown Swiss (BS), Jersey (JE) and Holstein (HO) populations

**Genotypic and allelic frequencies**	**BS**	**JE**	**HO**
11	0.4269	0.4305	0.4065
12	0.2582	0.2652	0.3038
22	0.3149	0.3043	0.2897
Major allele frequency	0.5558	0.5631	0.5585
Minor allele frequency	0.4442	0.4369	0.4415
Percentage of loci with fixed major allele	7.9043	8.7264	5.8522

**Figure 2:**
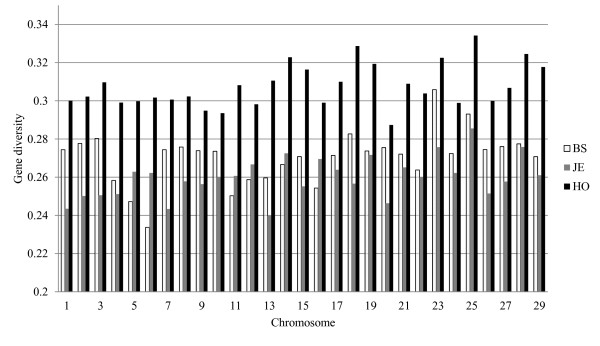
Average gene diversity expressed in terms of observed heterozygosity per chromosome in Brown Swiss (BS = white bar), Jersey (JE = grey bar) and Holstein bulls (HO = black bar).

**Figure 3:**
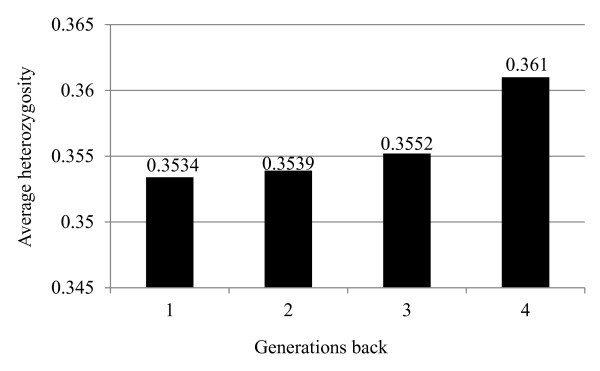
Average Heterozgosity for the last four generations traced back in the pedigree (generation interval = 5) in the pedigree of Holstein bulls.

Average gene diversity over all loci, per chromosome, in BS, JE and HO, expressed in terms of H_o_, are shown in Figure 2. Holsteins showed consistently higher H_o_ than JE and BS across all chromosomes. BS and JE had similar overall H_o_, however, depending on the chromosome, one or another of the two breeds had higher H_o_. Higher H_o_ for HO than BS and JE and similar overall H_o_ for BS and JE is consistent with the effective population sizes of this three breeds, which is higher for HO and lower and similar for BS and JE [3] Moreover, average heterozygosity of Holsteins showed a declining trend over the last four generations considering the generation interval of 5 years (Figure 3). Accordingly, H_o_ in HO has reduced from 0.361, when 4 generations were traced back in the pedigree, down to 0.3534, when one generation was traced back.

**Figure 4:**
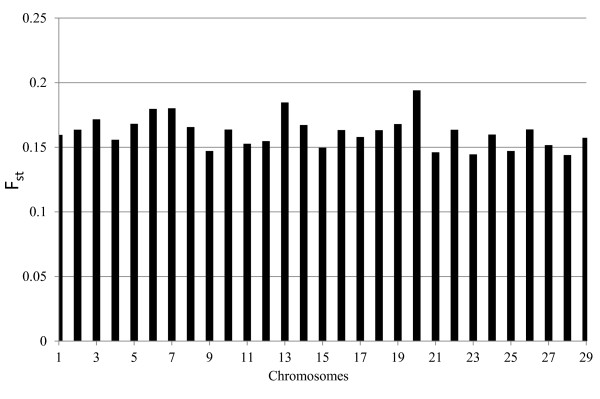
**Population genetic differentiation of the three breeds (Brown Swiss, Jersey and Holstein) measured by F**_**st**_.

Population genetic differentiation of BS, JE and HO, as measured by F_st_ (Figure 4) showed that the breeds are genetically differentiated at each chromosome. For example, the average measure of F_st_ in an ideally amalgamated population of BS, JE and HO on Chromosome 18 showed that the breeds are differentiated with an average value of F_st_ equal to 0.16. Higher value of F_st_ indicates the presence of higher genetic differentiation between subpopulations, which implies that pairs of genes between individuals within subpopulations are more related than those of individuals between subpopulations. The F_st_ values between each pair of populations indicated that BS vs. HO population has higher genetic differentiation than the other pairs (Table 2). However, the F_st_ values among the last four generations in the HO were below 0.1, suggesting that there was no considerable genetic differentiation in the HO bull population in the last four generations (data not shown). This may indicate the fact that there has not been new outbred genetic material introduced to the bull population over the last four generations, except the use of commonly used sires of good genetic merit over generations. The relatedness between individuals within breed in BS vs. HO populations relative to the total population was higher (0.28) than that in BS vs. JE (0.23) and JE vs. HO (0.22), which also implies that BS and HO gene pools are more differentiated compared to the other pairs of breeds.

**Table 2:** **Allelic richness (AR), F**_**st**_**index (subpopulation differentiation), frequency of private alleles (FPA, i.e. population-specific alleles) in each pair of ideally amalgamated populations and effective number of migrants (N**_**m**_**)**

**Breeds**	**AR**	**F**_**st**_	**FPA**	**N**_**m**_
BS and JE	1.874	0.155±0.002	0.056	0.47
BS and HO	1.899	0.196±0.002	0.082	0.32
JE and HO	1.912	0.142±0.002	0.079	0.53

A summary of allelic richness, average fixation indices, frequency of private alleles per breed pair are presented in Table 2. Higher frequency of private alleles (alleles that are present in one of the breeds, but not in another) was observed in BS vs. HO followed by JE vs. HO and BS vs. JE populations. This result is also in agreement with population differentiation results, as measured by F_st_ values. In addition, indirect estimates of gene flow indicated the presence of higher effective number of migrants (N_m_) between populations of JE and HO followed by BS and JE, while BS and HO populations showed the least N_m_. This might be one explanation for the higher values of population differentiation measures, in particular higher F_st_ between BS and HO.

**Figure 5:**
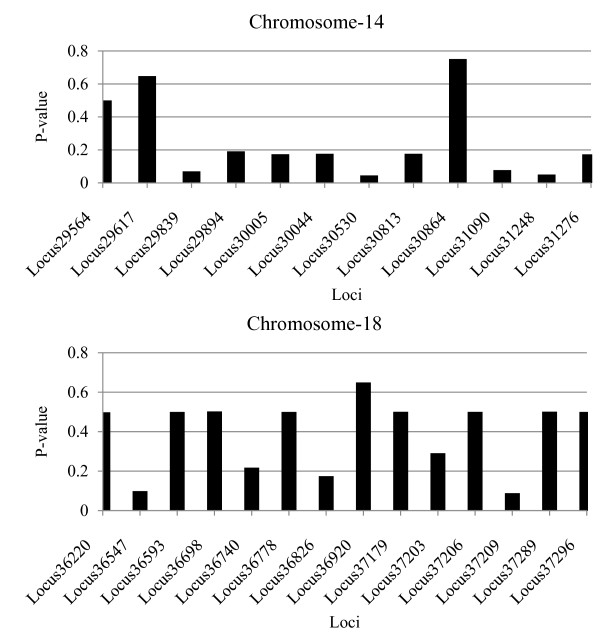
Loci with alleles drawn from the same distribution in all the three breeds (Brown Swiss, Jersey and Holstein) on Chromosome 14 and 18.

The exact-test for population differentiation of each breed pair across all loci showed highly significant differences among breeds regarding the distributions from which the alleles and genotypes were drawn from. Accordingly, the majority of loci have alleles or genotypes drawn from different distributions in the three breeds. However, there are some loci with alleles or genotypes drawn from the same distribution in all the breeds. For example, loci with alleles drawn from the same distribution in BS, JE and HO are shown for Chromosome 14 and 18 (Figure 5). This implies that alleles of those loci may not have been differentiated by selection, drift and inbreeding in the three bull populations. Moreover, the comparison of each pair of the three breeds with respect to the origin of their alleles is also presented. Accordingly, on average, alleles of 7.8, 5.5 and 3.1% of loci could be drawn from the same distribution in JE vs. HO, BS vs. JE and BS vs. HO populations, respectively (Table 3). Similar results were obtained for the percentage of loci with genotypes drawn from the same distribution (Table 4).

**Table 3:** **Percentage of loci in each pair of populations in which the null hypothesis that alleles are drawn from the same distribution in all populations has been accepted (*p*** **> 0.05) along with the corresponding average p-values, as a measure of population differentiation**

**Chromosome**	**BS vs. JE**	**p-value**	**JE vs. HO**	**p-value**	**BS vs. HO**	***p*-value**
1	5.603	0.3471	8.191	0.359	3.228	0.345
2	4.890	0.3367	7.316	0.345	3.658	0.396
3	5.569	0.3475	7.886	0.393	3.049	0.333
4	5.547	0.3155	8.178	0.395	3.239	0.339
5	6.436	0.3239	6.436	0.396	3.644	0.34
6	5.030	0.3423	6.157	0.358	2.897	0.334
7	5.629	0.3166	6.819	0.388	3.066	0.317
8	4.807	0.3475	7.579	0.381	3.378	0.330
9	6.891	0.3896	7.243	0.368	3.773	0.348
10	5.152	0.3454	7.366	0.403	2.985	0.312
11	5.037	0.3898	6.932	0.381	3.420	0.313
12	5.526	0.3698	7.027	0.389	3.483	0.301
13	4.485	0.3478	6.232	0.379	2.912	0.321
14	6.048	0.3748	7.046	0.368	2.466	0.316
15	5.215	0.4109	8.344	0.355	3.313	0.332
16	5.802	0.3395	7.735	0.407	3.244	0.388
17	5.381	0.3859	7.612	0.382	2.822	0.386
18	5.329	0.3559	6.191	0.381	3.683	0.384
19	3.364	0.3772	7.645	0.375	3.211	0.372
20	6.009	0.3741	6.989	0.326	3.005	0.321
21	4.822	0.3203	8.160	0.387	3.932	0.344
22	5.412	0.3059	7.512	0.364	4.281	0.322
23	4.926	0.3519	8.768	0.341	2.365	0.395
24	5.276	0.3356	8.198	0.405	2.110	0.434
25	5.038	0.3228	7.996	0.391	1.972	0.333
26	9.022	0.3509	8.072	0.381	2.374	0.478
27	4.487	0.3763	17.735	0.380	3.098	0.393
28	6.037	0.3634	7.794	0.339	2.305	0.368
29	5.865	0.3257	7.820	0.365	3.226	0.333
**Average**	**5.470**	**0.351**	**7.827**	**0.375**	**3.108**	**0.353**

**Table 4:** **Percentage of loci in each pair of populations in which the null hypothesis that genotypes are drawn from the same distribution in all populations has been accepted (*p*** **> 0.05) along with the corresponding average p-values, as a measure of population differentiation**

**Chromosome**	**BS vs. JE**	**p-value**	**JE vs. HO**	**p-value**	**BS vs. HO**	***p*-value**
1	7.217	0.338	8.191	0.080	4.903	0.369
2	7.577	0.309	6.980	0.099	5.151	0.398
3	7.642	0.365	7.886	0.108	4.472	0.377
4	7.206	0.331	17.085	0.143	4.575	0.363
5	8.140	0.346	6.436	0.163	5.395	0.371
6	6.962	0.357	6.157	0.188	4.185	0.361
7	8.238	0.336	6.819	0.205	3.982	0.308
8	7.016	0.357	7.579	0.225	5.154	0.363
9	8.903	0.374	7.243	0.254	5.483	0.371
10	8.089	0.346	7.366	0.287	4.959	0.315
11	6.654	0.386	6.932	0.321	5.129	0.355
12	7.447	0.383	7.027	0.351	5.646	0.335
13	6.465	0.361	6.814	0.383	4.368	0.353
14	7.575	0.370	7.046	0.415	3.934	0.311
15	7.055	0.404	8.282	0.446	4.663	0.361
16	8.110	0.388	7.798	0.486	4.554	0.404
17	7.349	0.392	7.677	0.521	5.249	0.357
18	6.897	0.365	6.191	0.556	4.232	0.392
19	5.963	0.419	7.645	0.596	5.122	0.365
20	7.120	0.408	6.989	0.627	4.311	0.333
21	7.122	0.306	8.160	0.665	5.935	0.386
22	7.027	0.342	7.512	0.682	5.654	0.418
23	6.995	0.381	8.768	0.710	4.729	0.334
24	6.818	0.381	8.198	0.748	3.247	0.453
25	7.229	0.374	7.886	0.781	3.614	0.270
26	8.072	0.336	8.547	0.818	3.799	0.412
27	8.974	0.364	7.799	0.854	4.167	0.426
28	9.330	0.355	8.342	0.895	4.281	0.324
29	7.234	0.427	7.722	0.901	5.279	0.333
**Average**	**7.463**	**0.365**	**7.830**	**0.466**	**4.696**	**0.363**

The amalgamated BS vs. HO population showed the highest average inter-population gene differentiation both dependent (D_st_ = 0.03) and independent (D_st_’ = 0.07) on the number of subpopulations, and also the highest expected total heterozygosity (Y_t_ = 0.33) compared with the ideally amalgamated populations of BS vs. JE, or JE vs. HO. Similarly, the highest G_st_ and G_st_’ (12.5 and 19.7%, respectively) were also observed in an ideally amalgamated population of BS vs. HO. Therefore, the measure of inter-population gene differences also revealed that BS vs. HO population had the highest genetic differences compared to the other two pairs of breeds (BS vs. JE and JE vs. HO). Overall, the results indicated higher genetic diversity in an ideally amalgamated population of BS vs. HO.

## Discussion

The recent decline in diversity is sufficiently rapid that loss of diversity should be of concern to animal breeders [23]. Several authors e.g. [24] demonstrated different models to describe deviations from Hardy-Weinberg proportions. The exact test for Hardy-Weinberg disequilibrium [9,25] within breeds showed a significant deviation in each breed in this study (*P* < 0. 01). The populations also showed several loci with a significant heterozygote deficit (*P* < 0.01) but no loci with significant heterozygote excess, which implies the application of genetic selection and inevitably the role of random genetic drift and inbreeding in each breed. Generally, the results have showed that there are some loci (from 3.1 to 7.8%) with alleles drawn from the same distribution in all the populations. This may suggest the fact that, over time and through forces like selection and random genetic drift, the allele frequencies have been largely changed in the breeds, where very little of the original genomes are preserved.

Each breed showed considerable difference between the observed and expected number of heterozygous individuals across loci. However, in the ideally amalgamated pairs of the populations, the difference between the observed and expected number of heterozygous individuals appears to be smaller suggesting that crossbreeding could be carefully considered for increasing diversity in the future if needed. In livestock species, heterozygote deficiencies can be interpreted as the consequence of many factors, such as selection, population subdivision, or inbreeding [26].

Populations are said to be undifferentiated if F_st_[27]. In this study each pair of breeds showed higher values, which implies that the populations have different gene pools. However, F_st_ among the last four generations in HO was below 0.1, suggesting that there has been no significant introduction of more outbreed gene pool into the HO population over the last four generations. These results imply measures of population differentiation based on F_st_ have been described as reliable. For example, pair-wise F_st_ values were significantly correlated between bi-allelic loci and microsatellite datasets in Atlantic salmon, and similar result was found with regard to the overall heterozygosity [27].

The highest proportion of total genetic variation attributed to between breed differentiation was observed in BS vs. HO. The proportion of between breed genetic variation observed in this study was comparable to the average between breed variation (7.03%) reported in nine populations of Argentinean Creole cattle populations [28]. Studies in the past demonstrated that Wright’s F_st_ results were reliable and most consistent with Reynold’s distances, Nei’s minimum distance measures and eight other genetic distance measures for ordering populations, which are widely used and well-established measures of genetic differentiation [29]. In this study, the mean F_st_ indicated that BS vs. HO population has higher genetic diversity than BS vs. JE and JE vs. HO ideally amalgamated populations. The average estimates, based on microsatellites, of F_st_ in 20 Northern European cattle breeds was 0.11 ± 0.01 [30], which is comparable with the findings in this study.

To summarize, Brown Swiss, Jersey and Holstein bull populations have substantially different gene pools. An interesting result was the heterozygote deficit observed in each of the populations in this study. In livestock species, heterozygote deficiencies can be explained by several factors, such as selection, population subdivision, drift and inbreeding. Each breed showed a considerable difference between the observed and expected number of heterozygous individuals across loci. However, in the ideally amalgamated pairs of the populations, the difference between the observed and expected number of heterozygote individuals across loci appears to be smaller, suggesting that crossbreeding could be carefully considered for increasing diversity if needed in the future. At the present level of genetic diversity, crossbreeding is not a necessity, however if loss of genetic diversity within each breed worsens in the future, crossing can be considered as an option to increase total genetic diversity within breeds.

## Conclusions

The results suggested that the within population genetic diversity accounts for a higher proportion of the total genetic diversity in ideally amalgamated populations than the diversity between populations. The results of private alleles frequencies in this study indicated that each breed might contain unique genes or gene combinations that are absent in another breed. The study demonstrates that even with a much smaller population size, BS showed similar gene diversity to the Jersey breed, while Holstein showed higher gene diversity than both breeds in agreement with their reported effective population sizes. BS and HO seem to have higher population differentiation (F_st_) compared to the other pairs (BS vs. JE and JE vs. HO). If BS and HO were to be amalgamated, higher total expected gene diversity would be obtained as compared to the other pairs of breeds (BS vs. JE and JE vs. HO). If the loss of genetic diversity within breeds worsens in the future, the use of crossbreeding might be an option to recover genetic diversity, especially for the breeds with small population size.

F_is_ is the average over all subpopulations of the correlation between uniting gametes relative to those of their own subpopulation. F_st_ is the correlation between random gametes within subpopulations relative to gametes of the total population, which is a measure of subpopulations differentiation. F_it_ is the correlation between uniting gametes that generated the individual relative to gametes of the total population; subscripts is, st and it stand for individual relative to subpopulation, subpopulation relative to total population, and individual relative to total population, respectively.

## **Abbreviations**

BS = Brown Swiss; JE = Jersey; HO = Holstein; HWE = Hardy-Weinberg Equilibrium.

## Authors’ Contributions

MGM conducted the analyses and wrote the manuscript. FSS supervised the study and critically reviewed the manuscript. All authors read and approved the final manuscript.

## Competing interests

The authors declare that they have no competing interests.
